# Roles of Piwi Proteins in Transcriptional Regulation Mediated by HP1s in Cultured Silkworm Cells

**DOI:** 10.1371/journal.pone.0092313

**Published:** 2014-03-17

**Authors:** Tsuneyuki Tatsuke, Li Zhu, Zhiqing Li, Hitoshi Mitsunobu, Kaito Yoshimura, Hiroaki Mon, Jae Man Lee, Takahiro Kusakabe

**Affiliations:** 1 Laboratory of Silkworm Science, Kyushu University Graduate School of Bioresource and Bioenvironmental Sciences, Fukuoka, Japan; 2 Department of Biological Chemistry and Molecular Pharmacology, Harvard Medical School, Boston, Massachusetts, United States of America; Ludwig-Maximilians-Universität München, Germany

## Abstract

Piwi proteins are part of a superfamily of Argonaute proteins, which are one of the core components of the RNA silencing pathway in many eukaryotes. Piwi proteins are thought to repress the transposon expression both transcriptionally and post-transcriptionally. Recently, *Drosophila melanogaster* Piwi was recently reported to associate with chromatin and to interact directly with the Heterochromatin Protein 1 (HP1a). However, similar interactions have not been reported in other higher eukaryotes. Here we show that silkworm Piwi proteins interact with HP1s in the nucleus. The silkworm, *Bombyx mori*, has two Piwi proteins, Ago3 and Siwi, and two typical HP1 proteins, HP1a and HP1b. We found that HP1a plays an important role in the interaction between Ago3/Siwi and HP1b in the ovary-derived BmN4 cell line. We also found that Ago3/Siwi regulates the transcription in an HP1-dependent manner. These results suggest that silkworm Piwi proteins function as a chromatin regulator in collaboration with HP1a and HP1b.

## Introduction

RNA silencing triggered by 20–35-nucleotide (nt) small RNA is involved in the regulation of gene expression both transcriptionally and post-transcriptionally [1]–[3]. The RNA silencing system in the fission yeast, *Schizosaccharomyces pombe*, is particularly well characterized [2], [3]. In *S. pombe*, which does not have Piwi-interacting RNAs (piRNAs), small interfering RNAs (siRNAs) and Argonaute 1 (Ago1) recognize nascent transcripts and assemble centromeric heterochromatin by recruiting the Heterochromatin Protein 1 (HP1) homolog Swi6 [2], [4]. In higher eukaryotes, multiple Argonaute proteins have been found and divided into two subfamilies [5]. The Piwi subfamily proteins, part of the superfamily of Argonaute proteins, are also thought to have an important role in the regulation of heterochromatin and chromatin maintenance [1], [6]–[8]. However, the silencing mechanism mediated by Piwi proteins has not been well understood.

*Drosophila melanogaster* Piwi (dPiwi) was recently reported to associate with chromatin and to interact directly with *D. melanogaster* HP1a (dHP1a). Thus, it is possible that dPiwi silences transcription by heterochromatin assembly analogous to the case in *S. pombe* [7]–[9]. HP1 has been generally known to be a non-histone chromosomal protein that plays diverse and critical roles in chromatin structure, transcription, DNA replication, chromosome segregation, and genomic stability [3]. The interaction between dPiwi and dHP1a is mediated by a chromoshadow domain in dHP1a and a PxVxL-type motif in dPiwi [9]. In addition to the direct interaction between dPiwi and dHP1a, dPiwi has been reported to guide epigenetic factors to their target sites and to be required for the transcriptional silencing of transposons in *Drosophila* [7], [8].

The silkworm, *Bombyx mori*, has two Piwi genes encoding Ago3 and Siwi, and two typical HP1 proteins, HP1a and HP1b, in the genome [10]. Similarly to *Drosophila*, silkworm Ago3 and Siwi play an important role, at least in part, in repressing transposons [11], [12]. We previously reported that silkworm HP1a forms a homo- and heterodimer with HP1b and interacts more strongly with Su(var)3–9 than does HP1b, whereas HP1b has higher repression activity than HP1a [10]. Although direct interaction between Piwi and HP1a proteins has been reported in *Drosophila*, similar interactions have not been reported in other higher eukaryotes [9], to our knowledge. Here, we report the functional relationship between Piwi proteins and HP1 in the silkworm ovary-derived cultured cell line BmN4. The silkworm Piwi proteins were found to interact with HP1b through the mediation of HP1a, and Ago3/Siwi was shown to be involved in transcriptional regulation in an HP1-dependent manner. These findings suggest that Ago3/Siwi may function as the chromatin regulator in silkworms.

## Results and Discussion

### Silkworm Piwi Proteins Interact with HP1 in Nucleus

**Figure 1:**
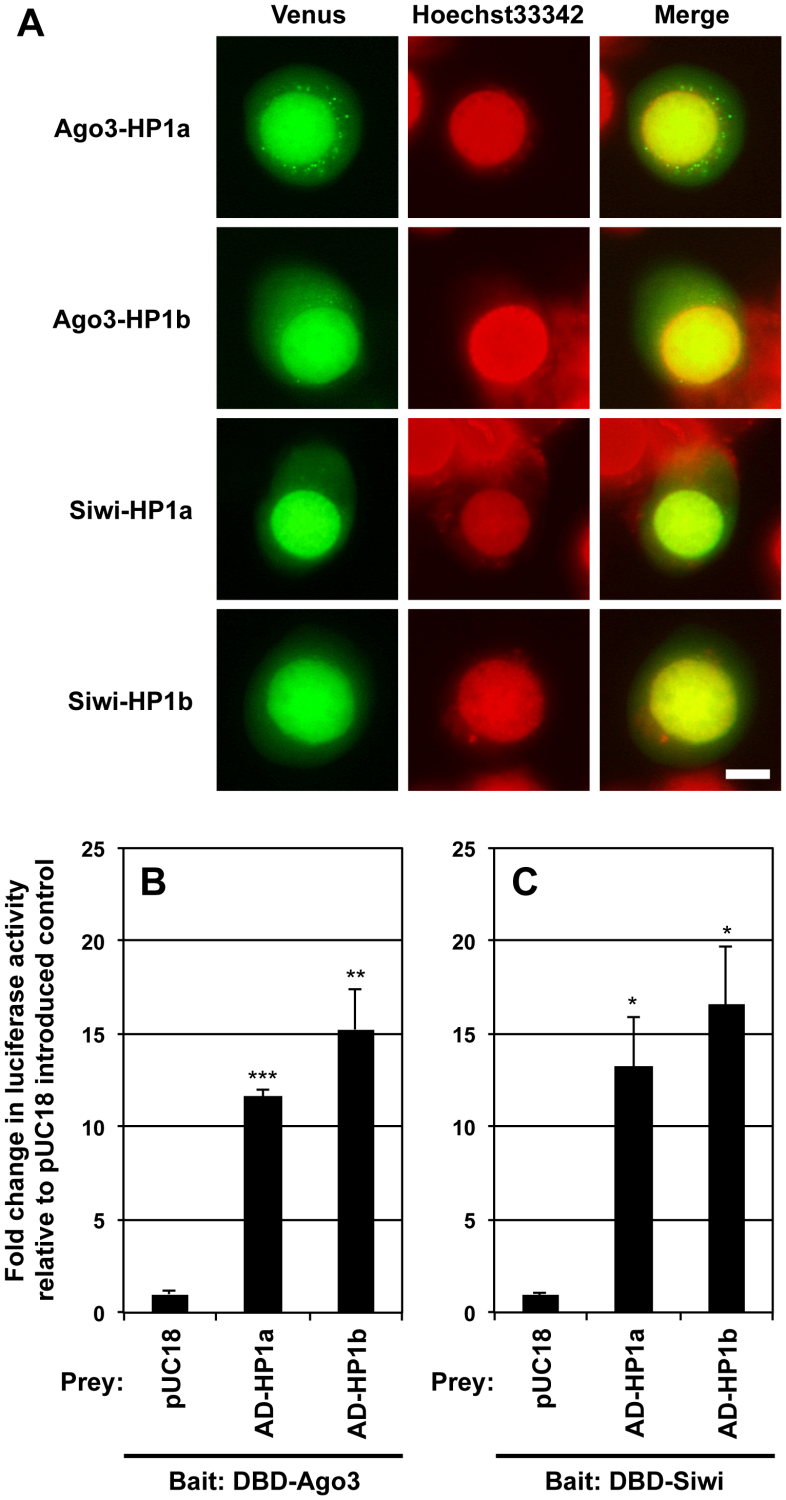
Silkworm Piwi proteins interacted with HP1a and -b in cultured silkworm cells. (A) The BiFC analysis of the interaction between *B. mori* Piwi and HP1 proteins in the silkworm cultured cell line BmN4. Venus and Hoechst33342 fluorescences indicate their localization and nucleus, respectively. Scale bar: 10 μm. (B, C) The insect two-hybrid (I2H) assay of the interaction between silkworm Piwi and HP1 proteins. BmN4 cells were transfected with 4×UAS-TATA-Luc reporter construct, along with expression vectors for GAL4 DNA-binding domain (DBD) and p65 activation domain (AD) fused to Ago3, Siwi, HP1a or HP1b as indicated. Empty cloning vector pUC18 was introduced as the control prey instead of pAD-HP1a or HP1b. Luciferase activities were measured at 72 h post-transfection. Values are relative luciferase activities in the pUC18-introduced control. Error bars = standard deviation (SD). The SDs and *P*-values are based on n = 3, and were determined by the *t*-test in comparison with the luciferase activities in the pUC18-introduced controls. *P<0.05, **P<0.01, ***P<0.001.

To test the direct interaction between dPiwi and dHP1a proteins in the silkworm ovary-derived cultured cell line BmN4, we employed BiFC (bimolecular fluorescence complementation; [13]) and analyzed the proteins’ localization. The signal of dPiwi-dHP1a interaction was observed primarily in the nucleus of the BmN4 cells (Figure S1A), whereas the EGFP-fused dPiwi was diffused in BmN4 cells as opposed to *Drosophila* cells ([14]; Figure S1B). Using this BiFC assay system, we examined the interactions of two silkworm Piwi-subfamily proteins, Ago3 and Siwi, with two canonical silkworm HP1 proteins, HP1a and -b, in BmN4 cells. As shown in Figure 1A, both Ago3 and Siwi interacted with both HP1a and HP1b primarily in the nucleus of the BmN4 cells. Interestingly, the interaction signals were also observed in tiny cytoplasmic granule-like structures in a certain number of BmN4 cells. Previously, we found similar Ago3 or Siwi granules in BmN4 cells [11], but it is not yet clear whether the granule-like structures observed in the present study are the same as those observed previously.

Unfortunately, a co-immunoprecipitation assay could not verify these interactions in BmN4 cells (data not shown), suggesting that silkworm Piwi interacts loosely or transiently with HP1 proteins. Thus, we carried out an insect two-hybrid (I2H) analysis, a method with high sensitivity for the detection of this weak protein-protein interaction [15]. Similarly to the results obtained with BiFC, the co-expression of DBD-Ago3 or -Siwi (as bait) with either of the two AD-HP1 proteins (as prey) increased the reporter activity more than 10-fold in BmN4 cells (Figure 1B and C). The I2H system uses the GAL4 DNA-binding domain (GAL4 DBD) with a typical nuclear localization signal (NLS); thus, there remains the possibility that the DBD-mediated nuclear import of silkworm Piwi proteins may lead to false-positive results for the interaction between silkworm Piwi and HP1 proteins.

To exclude this possibility, we constructed another I2H system in which TetR without typical NLS and tetO sequences was used instead of the GAL4 DBD and UAS sequences, respectively. In agreement with the results obtained with the GAL4-based I2H, the reporter activity of the TetR-based I2H analysis indicated the interaction between silkworm HP1 and Piwi-subfamily proteins (Figures S1C and D). Taken together, these results clearly indicate that silkworm Piwi proteins interact with two canonical HP1 proteins in the nucleus of BmN4 cultured cells.

In *Drosophila*, dimeric dHP1a binds to a PxVxL-type motif in the N-terminal domain of dPiwi, which is found only in the *Drosophila* Piwi protein, through its C-terminal chromoshadow domain [9]. To investigate which region of the Piwi protein interacts with HP1s, i.e., the N- or C-terminal domain, we performed I2H analyses between split silkworm Piwi and HP1 proteins (Figure S2). In all silkworm Piwi proteins and dPiwi undergoing the I2H assay with HP1 proteins, the N-terminal domain showed stronger luciferase activities than the C-terminal domain (Figure S3). Thus, similar to the case in *Drosophila*, these results suggest that the N-terminal domains of Piwi have a major contribution to the interaction with HP1a/b in silkworm BmN4 cells.

The silkworm genome sequence database, however, revealed that Ago3 and Siwi do not contain PxVxL-type motifs in their amino acid sequence, suggesting that the silkworm Piwi-subfamily proteins interact with HP1s at regions distinct from the PxVxL-type motif, unlike other canonical HP1 interactors [16]. Additionally, the interaction of the N-terminal domain of Piwi proteins with HP1s was stronger than that of full-length Piwi with HP1s, even in the case of dPiwi-dHP1a interaction (Figure S3). This result may indicate that the potential high affinities of N-terminal domains for HP1s are modulated by other regions of the Piwi proteins. These results thus suggest the possibility that Piwi proteins of other species lacking typical HP1 interaction motifs can interact with HP1 in the same way as silkworm Piwi proteins.

### HP1a Mediates the Interaction between Silkworm Piwi Proteins and HP1b

**Figure 2:**
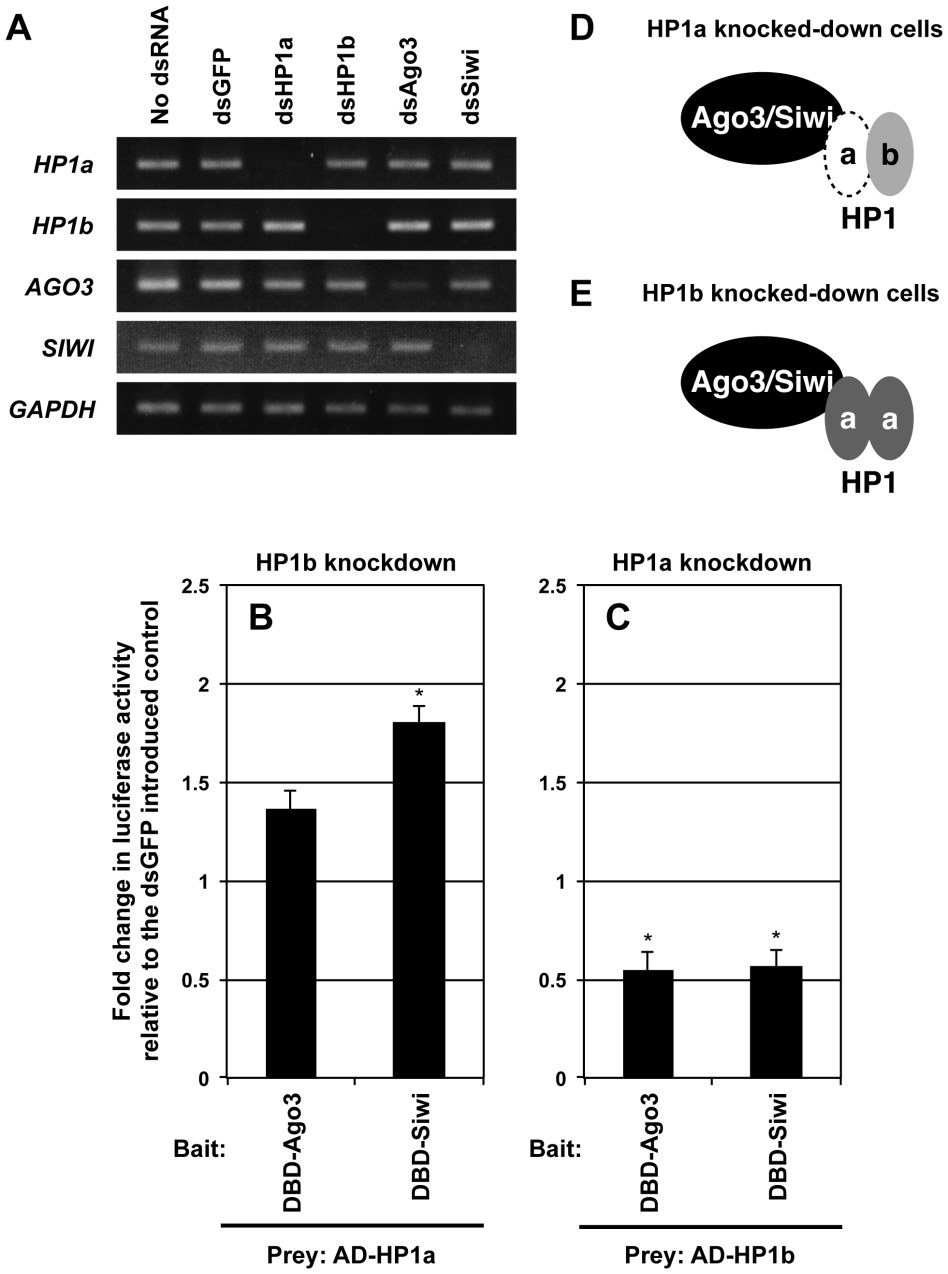
HP1a is required for the interaction between silkworm Piwi proteins and HP1b. (A) Semi-quantitative RT-PCR analyses were performed using the cDNA library from Ago3-, Siwi-, HP1a- or HP1b-gene-knockdown BmN4-SID1 cells. RNAi-induced BmN4-SID1 cells showed a reduction of target gene expression level 72 h after dsRNA introduction. (B, C) The I2H assay of the interaction between silkworm Piwi and HP1 proteins in each HP1-depleted BmN4-SID1 cell. At 72 h after the introduction of dsHP1a, dsHP1b or dsGFP (control), BmN4-SID cells were transfected with 4×UAS-TATA-Luc reporter construct, along with expression vectors for GAL4 DNA-binding domain (DBD) and p65 activation domain (AD) fused to Ago3, Siwi, HP1a or -b as indicated. Luciferase activities were measured at 72 h post-transfection. Luciferase activities were normalized to that of dsGFP-introduced controls (n = 3). Error bars = SD. The SDs and *P*-values (*t*-test, *P<0.05) are based on n = 3, and are based on comparisons with luciferase activities in dsGFP-introduced controls. (D, E) A model for the interaction between silkworm Piwi and HP1 proteins in each HP1-gene-knockdown BmN4-SID1 cell.

Since silkworm HP1a and HP1b have been demonstrated to form a heterodimeric complex [10], we analyzed the mutual dependency of HP1s in the interaction with Ago3 and Siwi. Using BmN4-SID1 cells, a cell line highly sensitive to soaking RNAi [17], we performed I2H analyses under knockdown conditions of each HP1 protein. Semi-quantitative RT-PCR analyses clearly demonstrated the specific knockdown of HP1 mRNAs by RNAi (Figure 2A). Compared to the dsGFP-treated cells used as a control, the interaction of HP1b with Ago3 or Siwi was decreased in the absence of HP1a (Figure 2C), suggesting that the interaction between Ago3/Siwi and HP1b is mediated by HP1a (Figure 2D).

In contrast, the interaction of HP1a with Ago3 or Siwi detected by I2H was increased in BmN4 cells with down-regulated HP1b expression (Figure 2B). As illustrated in Figure 2E, the increased HP1a homodimer formation in the absence of HP1b would enhance its interaction with Ago3 and Siwi, and the exclusion of the HP1a/b heterodimer would remove the negative transcriptional activity of HP1b.

We previously reported that the silkworm Ago3 and Siwi co-localize in nuage-like cytoplasmic granules of BmN4 cells [11]. In *Drosophila*, two piRNA-related cytoplasmic granules, nuages in nurse cells and Yb bodies in ovarian somatic cells (OSCs), have been reported [14], [18]. In nurse cells, the localization of piRNA-related proteins to nuages depends on Vasa, a marker for germ cells [18]. In contrast, Armitage and Yb, the Yb body components, are required for the entry of dPiwi into the OSC nucleus [14]. We thus examined the effect of depletion of these piRNA-related genes on the interaction between Ago3/Siwi and HP1a/b. As nuage-related genes, we used *SPN-E* and *TUD* in addition to VLG (Vasa-like gene; [19]). There were weak but not significant effects in the interaction between Ago3/Siwi and HP1a/b under knockdown conditions (Figures S4 and S5), suggesting that Ago3 and Siwi interact with HP1a independently of other piRNA-related genes. Only the Siwi-HP1a interaction was increased significantly in VLG knockdown cells (Figure S5C).

**Figure 3:**
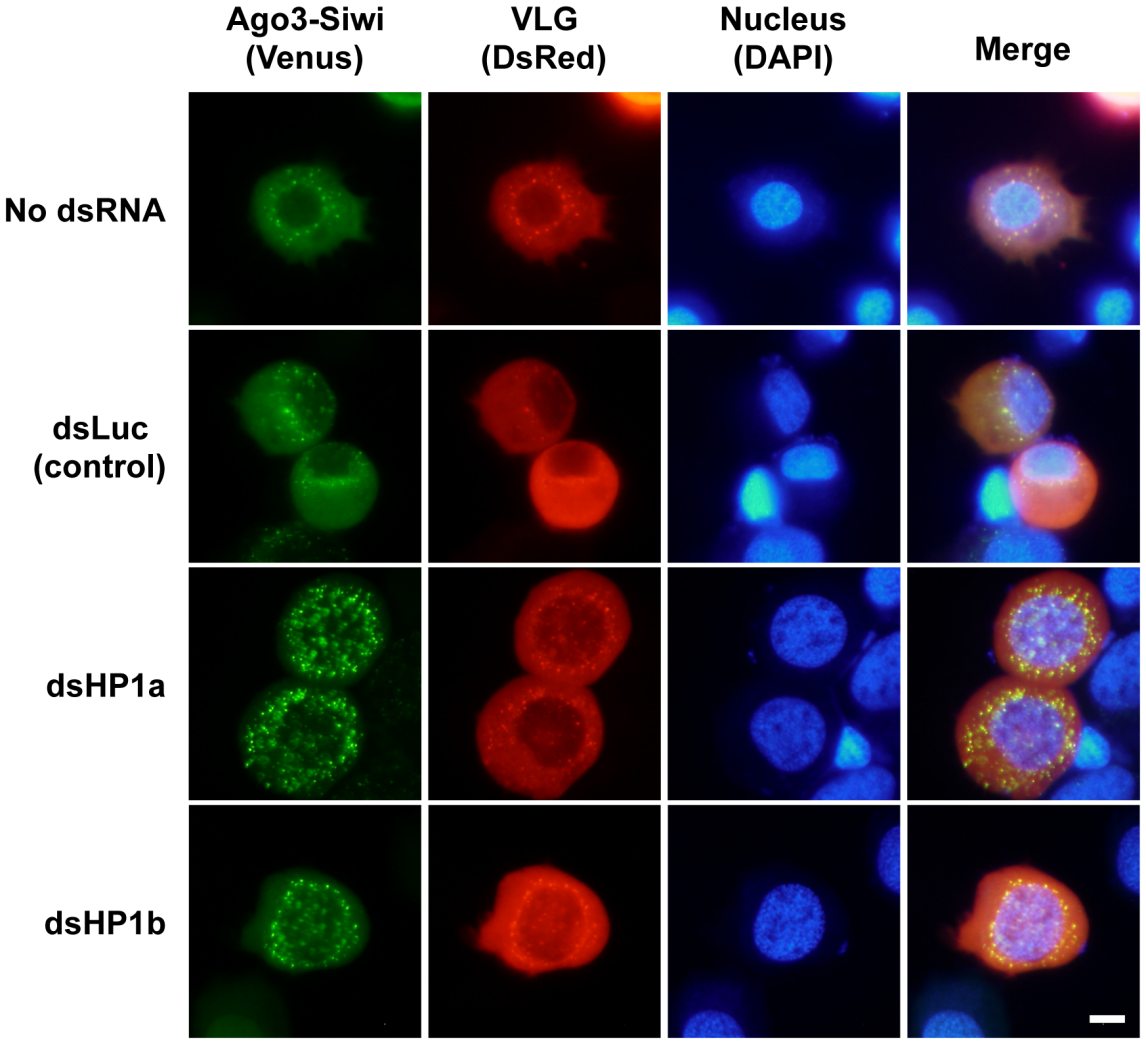
The Ago3-Siwi signal in nuage-like granules increased under the HP1a or -b knocked-down conditions in the silkworm cultured cells. The BiFC analyses for the interaction between Ago3 and Siwi proteins in each HP1-gene-knockdown BmN4-SID1 cell. The BiFC analyses were performed at 72 h after the introduction of dsHP1a, dsHPb or dsLuc (control). Venus, DsRed and 4′,6-diamidino-2-phenylindole (DAPI) fluorescences indicate Ago3-Siwi, VLG (as a marker for nuage) and the nucleus, respectively. Scale bar: 10 μm.

In agreement with our previous findings [19], the present BiFC analysis detected the interaction of Ago3-Siwi in nuage-like granules, which are marked by the localization of DsRed-fused VLG (Figure 3). Interestingly, the Ago3-Siwi signal increased and became clearer under the HP1a or -b depletion conditions, suggesting that HP1 proteins stimulate the nuclear import of Ago3 or Siwi from nuage-like granules. Taken together, the past and present findings indicate that silkworm HP1s may compete or coordinate with VLG to determine the subcellular localization of Piwi proteins predominantly through Siwi-HP1a interaction.

### Roles of Silkworm Piwi Proteins in Transcription

**Figure 4:**
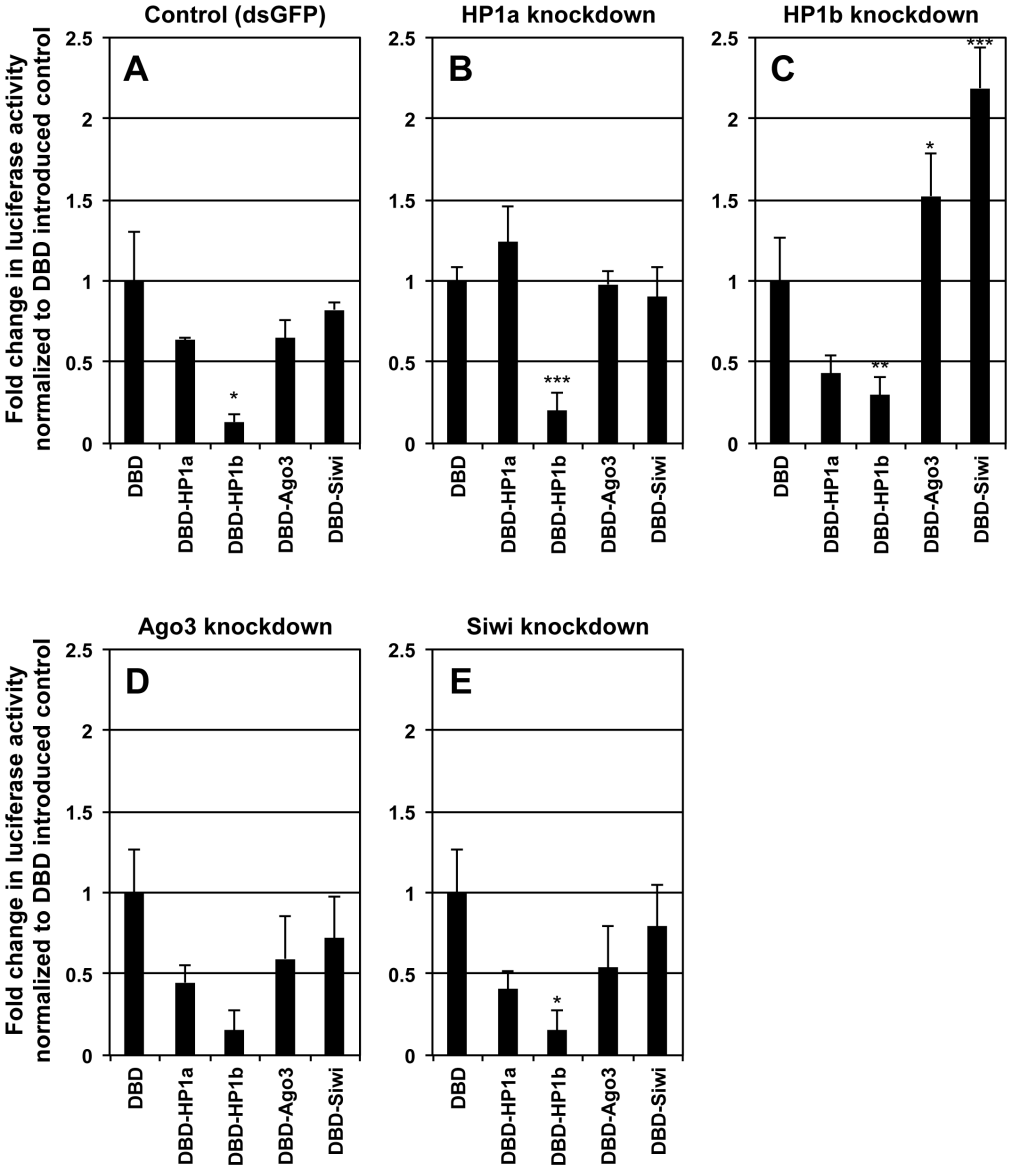
Silkworm Piwi proteins repressed transcription in an HP1-dependent manner. (A–F) Transcriptional repression of luciferase 2P (luc2P) reporter gene under the control of the *hsp* promoter by silkworm Piwi and HP1 proteins in BmN4-SID1 cells with dsRNA against Ago3, Siwi, HP1a or HP1b. The *hsp* promoter-based luc2P reporter contains five synthetic GAL4-UAS sites and was used in all transfections. BmN4-SID1 cells were transfected with a Luc2P reporter under the control of the *hsp* promoter, upstream of which five UAS were located for the *hsp* promoter. Cells were co-transfected with an expression vector for GAL4 DNA-binding domain (DBD)-fused Ago3, Siwi, HP1a or HP1b proteins at 72 h after dsRNA introduction. Luciferase activities were measured at 72 h post-transfection. Luciferase activities are represented as relative values based on DBD (empty plasmid introduced cells) as a standard. Error bars = SD. The SDs and *P*-values (determined by the t-test, *P<0.1, **P<0.05, ***P<0.01, which was checked by a comparison with the luciferase activities in the DBD-introduced controls with DBD-HP1a, DBD-HP1b, DBD-Ago3 or DBD-Siwi-introduced cells) are based on n = 3.

On the basis of the existence of a Piwi-HP1 interaction, Ago3/Siwi might contribute to transcriptional gene silencing (TGS) through the induction of heterochromatin formation as do *S. pombe* Ago1 and *D. melanogaster* Piwi [1], [2], [4], [6]–[9], [20]. To assess whether Ago3/Siwi could function as a transcriptional repressor, we performed a transcription repression assay using the GAL4-UAS-based assay system previously reported [10]. We found that GAL4 DBD-fused Piwi proteins could be forcibly recruited to UAS elements upstream of the reporter luciferase gene without guidance by piRNA [10]. As previously reported, HP1 proteins repressed the transcription, and the repression induced by DBD-HP1b was more efficient than that by DBD-HP1a. Compared to HP1 proteins, DBD-Ago3 and -Siwi have only modest repression activity (Figure 4A).

We next attempted to examine the repression activity of silkworm Piwi proteins in BmN4-SID1 cells under conditions in which HP1a, HP1b, Ago3 or Siwi were knocked down (Figure 4). The transcriptional repression mediated by DBD-Ago3 and -Siwi in the absence of HP1a was slightly decreased compared to that of the control (Figures 4A and B), suggesting that Ago3 and Siwi by themselves have no function in the transcriptional repression. Interestingly, DBD-Ago3 or -Siwi enhanced the transcription in the absence of HP1b (Figure 4C), implying that Ago3/Siwi has another interactor which regulates transcription positively and competes with HP1b in binding to Ago3/Siwi.

Indeed, it has been reported that small RNA machinery interacts with RNA polymerase II, the second-largest RNA subunit, and affects heterochromatic silencing in *D. melanogaster* and *S. pombe* [21], [22]. These findings suggest that HP1a mediates the interactions of Ago3/Siwi with both HP1b and a positive regulatory factor. Unlike the knockdown of HP1a or HP1b, the transcription-repression activities of DBD-HP1a and -HP1b were not affected by the presence or absence of silkworm Piwi genes (Figures 4D and E). In summary, we suspect that HP1a functions as a “hub” for the epigenetic chromatin regulation.

The chromatin-loading mechanism of silkworm Piwi proteins at their target loci has not yet been elucidated, but is thought to be mediated by piRNA as in the *S. pombe* system [4]. The HP1 proteins would be recruited to a target site in a sequence-specific manner guided by the Piwi-piRNA complex without histone modification, such as the typical heterochromatin maker H3K9 methylation [3]. At the target site, Piwi-HP1 complex functions as a rapid transcriptional repressor depending on the transcriptional repression activity of HP1 proteins before histone modifications against selfish genetic elements are established. Namely, the silkworm Piwi complex with an HP1a homodimer is a moderate transcriptional modulator and can recruit transcriptional activators or epigenetic factors, such as Su(var)3–9 [23]. At an early stage of the fist-time invasion of selfish genetic elements, the invasion site of which might be epigenetically naked and cannot be recognized by HP1 proteins, a certain amount of Piwi complex assembles with HP1a-HP1b heterodimers at the transposed selfish genetic elements guided by piRNA, represses their transcription and then forms a broad heterochromatic region by the ability of HP1 proteins to spread. This proposed mechanism might be the first step of *de novo* heterochromatin formation on selfish genetic elements.

## Materials and Methods

### Construction of Plasmids

The entry clones for dHP1a and dPiwi were constructed on pENTR11 from Invitrogen. To obtain the entry clone for dHP1a, the total RNA was isolated from the *Drosophila melanogaster* Schneider 2 cultured cell. This total RNA was reverse-transcribed with oligo (dT) primer and amplified with the primer set: 5′-GGGCC ATGGG CAAGA AAATC GACAA CCCTG-3′ and 5′-GGGCT CGAGT TAATC TTCAT TATCA GAGTA CCAGG-3′. The dPiwi flagment was amplified from pGEX-Piwi (kindly provided from Dr. Siomi) with the primer set: 5′-GGGCC ATGGC TGATG ATCAG GGACG TGGAC GCAGG-3′ and 5′GGGTC TAGAT TATAG ATAAT AAAAC TTCTT TTCG-3′. The primer sets were designed based on the registered sequence data. The amplified dHP1 and dPiwi cDNA were digested with *Nco*I and *Xho*I or *Xba*I and subcloned into between the *Nco*I site and the *Xho*I or *Xba*I site of the pENTR11. The resulting plasmids were named dHP1-pENTR11 and dPiwi-pENTR11, respectively.

N-terminal fusion DEST vectors, pIE2-DBD, pIE2-AD, pie2RW, pnVW or pcCW, which contains split Venus or Cerulean, respectively, were transcribed by the IE2 promoter from the *Orgyia pseudotsugata* nuclear polyhedrosis virus [15], [24]. The Gateway LR reaction between the entry vectors, BmVLG-pENTR11, BmHP1a-pENTR11, BmHP1b-pENTR11, BmAgo3-pENTR11 and Siwi-pENTR11, and the DEST vectors was performed using LR Clonase™ Enzyme Mix (Invitrogen) according to the manufacturer’s protocols recommended in manufacturer’s manual [10], [11], [25]. The resulting plasmids were pDBD-HP1a, pDBD-HP1b, pDBD-Ago3, pDBD-Siwi, pDBD-dPiwi, pDsRed-VLG, pCC-HP1a, pCC-HP1b, pCC-Ago3, pNV-Ago3, pNV-Siwi, pAD-HP1a and pAD-HP1b.

The construction of the TetR-based I2H plasmids, the split Piwi plasmids for I2H assay and dPiwi expression plasmids is described in Methods S1 and Table S1.

### Preparation of Double-stranded RNA

Double-stranded RNA (dsRNA) for *in vivo* RNAi reactions was prepared using the following procedures. For the dsRNA template, BmAgo3-545 and Siwi-587 fragments were amplified using primer pairs: 5′-TCTTC CAAGA GAAGG GTCAA GAAAT AG-3′ and 5′-CTGTC CCACT AGATA CGAGA GTTTG TG-3′; and 5′-CCTGA GTTGA TATAT CTAGT GCCAG AAC-3′ and 5′-TCATA CCTAT CAGCA TAATT CCTAG CC-3′, respectively, from BmN4 cDNA library and were inserted into a blunt-ended *Bst*BI-*Xba*I site of the pENTR11. The templates for *in vitro* transcription were synthesized via two-step PCR on the templates containing the HP1a, HP1b, BmAgo3-545 or Siwi-587 fragments using the primers, 5′-TAATA CGACT CACTA TAGGG TTTGT ACAAA AAAGC AGGC-3′ and 5′-TAATA CGACT CACTA TAGGG ACTTT GTACA AGAAA GCTGG-3′, for the first PCR and the primer, 5′-CAGTG AATTG TAATA CGACT CACTA TAGGG-3′, for the second PCR. The amplified DNA templates amplified were extracted with phenol/chloroform, precipitated with ethanol, and dissolved in H_2_O. Bi-directional transcription reactions were performed in the buffer (40 mM Tris-HCl, pH 7.5, 6 mM MgCl_2_, 10 mM DTT, 10 mM NaCl, 2 mM spermidine, 2 mM NTPs, 8 units of RNase inhibitor, and 20 units of T7 RNA polymerase), and incubated for 2 h at 37°C. The RNA products were purified and dissolved in 100 mM HEPES, pH7.0, incubated for 5 min at 94°C, and left to stand at room temperature for 30 min to allow the annealing of the two RNA strands, which are used as dsRNA solutions.

The preparation of dsRNAs against putative piRNA-related genes is described in Methods S1 and Table S1.

### Cell Culture and Transfection

The silkworm BmN4 cell line (a gift from Dr. Chisa Aoki, Kyushu University Graduate School) [11] and BmN4-SID1 transgenetic cell line (stored in our laboratory) [17] were maintained in IPL-41 medium (Gibco) with 10% fetal bovine serum at 27°C. These cells were split at a ratio of 1∶7 every 4–5 days, seeded in 24-well plates at a density of 5×10^4^ cells/well and, one day after seeding, overlaid with a lipid-plasmids complex (200 μl/well) in order to undergo transient transfection of plasmids. Shortly before the transfection, the lipid-plasmids complex was prepared by mixing 100 ng of each plasmids with 5 μl of PDD111 solution [26] in a final volume of 30 μl, followed by incubation at room temperature for 20 min and supplementation with 170 μl of Sf-900 II SFM (Gibco). The cells were incubated for 6 h, maintained for another 72 h in the medium replaced with 500 μl of IPL-41 and the cells were then collected for RNA extraction or microscopy. All the images were collected using a KEYENCE BZ-8000.

For RNAi experiments, BmN4-SID1 cells were used, and dsRNA for each target gene were added in the medium (100 ng/ml) 3 days before the transfection. To confirm RNAi efficiency for each target genes, *GAPDH*, *AGO3*, *SIWI*, *HP1a* and *HP1b*, the 5 primer pairs: 5′-GGCCG CATTG GCCGT TTGGT GCTCC G-3′ and 5′-GTGGG GCAAG ACAGT TTGTG GTGCA AGAAG-3′; 5′-TCAAT TGACT GGTTT AACAG ATGAT CAACG-3′ and 5′-CAAAC TTTCT TAATG GCCGC GTATC TGTCG-3′; 5′-ATGAC TCAGT ACTAT TCAAT CGTCA ATGCG-3′ and 5′-AACTT GGGAA GAGCA GTTGA AATAC AAGTG-3′; 5′-GGTAA AAAAG AGAAG AAAAC GGAGA CCAG-3′ and 5′-GGCTC GAGTT CTAAT TATTC AGATT CGGAT GG-3′; and 5′-GCCGA CAAGA AAAAA GAAAA TGAAC CAG-3′ and 5′-CCCTC GAGTC TTATT TAGTC TTCAT TATTC CC-3′, were used respectively. The amplified products were separated by electrophoresis through a 1% agarose gel in TAE buffer and stained with ethidium bromide.

### Cell Imaging

Fluorescence microscopy images were captured using Biozero BZ-8000 microscope (KEYENCE).

### Bimolecular Fluorescence Complementation Assay

Bimolecular fluorescence complementation (BiFC) analysis was based on the reassembling into a functional fluorescent protein though the association of protein fragments fused to the proteins of interest [13]. 100 ng of each expression plasmid for different silkworm Piwi or HP1 proteins fused to pnVW or pcCW, respectively, were co-transfected into the BmN4 cells.

### Luciferase Assay

Cells were harvested three days after transfection, washed with PBS and lysed with the lysis buffer (25 mM Tris-phosphate, pH 7.8, 2 mM DTT, 2 mM Trans-1, 2-diaminocyclohexane-N,N,N′,N′-tetraacetic acid monohydrate, 10% glycerol, 1% TritonX-100). The cell lysate was precipitated at 10,000 rpm for 1 min at 4°C. Luciferase activity was measured after mixing the resulting supernatant and luciferase substrate by ARVO (Perkin Elmer) using a luciferase reporter assay system (Promega).

## Supporting Information

Figure S1 **The interaction between Piwi and HP1 proteins in silkworm cultured cells.** (A) The interaction between *D. melanogaster* Piwi and HP1a in BmN4 cells. The BiFC analysis for the interaction between Piwi and HP1 proteins in the silkworm cultured cell line BmN4. Venus and Hoechst33342 fluorescences indicate their localization and nucleus, respectively. Scale bar: 10 μm. (B) The localization of EGFP-fused *Drosophila* Piwi protein in a BmN4 cell. EGFP and Hoechst33342 fluorescences indicate their localization and nucleus, respectively. Scale bar: 10 μm. (C, D) The insect two-hybrid (I2H) assay for the interaction between the silkworm Piwi and HP1 proteins with the use of the TetR-fused Piwi proteins instead of GAL4 DNA-binding domain. BmN4 cells were transfected with 9×tetO-IE2mini(L)-Luc reporter construct, along with expression vectors for TetR and p65 activation domain (AD) fused to Ago3, Siwi, HP1a or HP1b as indicated. Empty cloning vector pUC18 was introduced as the control prey instead of pAD-HP1a or HP1b. Luciferase activities were measured at 72 h post-transfection. Values are relative luciferase activities in the pUC18-introduced control. Error bars = SD. The SDs and *P*-values (determined by the *t*-test, **P<0.01, ***P<0.001, which was checked by comparing luciferase activities in pUC18-introduced controls with pAD-HP1a or -b-introduced cells) are based on n = 3.
(TIF)

Figure S2 **The split Piwi proteins for I2H baits.** Schematic structure of baits used in the split Piwi I2H assay. Piwi protein contains N, PAZ, MID, and PIWI domains. (A) dPiwiFL, residues 1–843; dPiwiNT, residues 1–491; and dPiwiCT, residues 492–843. (B) Ago3FL, residues 1–926; Ago3NT, residues 1–553; and Ago3CT, residues 554–926. (C) SiwiFL: 1–899; SiwiNT: 1–526; and SiwiCT: 527–899.
(TIF)

Figure S3 **The N-terminal domain of silkworm Piwi proteins interacts more strongly with HP1a/b than does the C-terminal domain.** (A–E) The I2H assay of the interaction between the split Piwi and HP1 proteins. BmN4 cells were transfected with 4×UAS-TATA-Luc reporter construct, along with expression vectors for GAL4 DNA-binding domain (DBD) and p65 activation domain (AD) fused to dPiwiFL, dPiwiNT, dPiwiCT, Ago3FL, Ago3NT, Ago3CT, SiwiFL, SiwiNT, SiwiCT, HP1a or HP1b as indicated. Luciferase activities were measured at 72 h post-transfection. Error bars = SD. The SDs and *P*-values (determined by the t-test, *P<0.1, **P<0.05, ***P<0.01, which was checked by comparing luciferase activities in cells transfected with DBD-fused N-terminal domain plasmids with that of DBD-fused C-terminal domain-transfected cells) are based on n = 3.
(TIF)

Figure S4 **The I2H assay for the interaction between the silkworm Piwi and HP1 proteins in each of silkworm Piwi gene knocked-down BmN4-SID1 cells.** (A, B) At 72 h after the introduction of dsAgo3, dsSiwi or dsGFP (control), BmN4-SID cells were transfected with 4×UAS-TATA-Luc reporter construct, along with expression vectors for GAL4 DNA-binding domain (DBD) and p65 activation domain (AD) fused to Ago3, Siwi, HP1a or -b as indicated. Luciferase activities were measured at 72 h post-transfection. The luciferase activities were normalized to that of dsGFP-introduced controls (n = 3 independent luciferase activities). Error bars = SD. *P*-values are shown in the bars. The SDs and *P*-values (*t*-test, which was checked by comparing luciferase activities in dsGFP-introduced controls with each of the dsRNAs against Ago3 or Siwi gene-introduced cells) are based on n = 2 (prey: AD-HP1b and bait: DBD-Ago3 in Siwi knocked-down BmN4-SID1 cells) and n = 3 (others).
(TIF)

Figure S5 **The I2H assay for the interaction between the silkworm Piwi and HP1 proteins in putative piRNA-related genes in knocked-down BmN4-SID1 cells.** (A–D) At 72 h after the introduction of each dsRNA against putative piRNA-related genes, dsVLG, dsSpnE, dsTud, dsArmi, dsYb or dsGFP (control), BmN4-SID cells were transfected with 4×UAS-TATA-Luc reporter construct, along with expression vectors for GAL4 DBD and p65 AD fused to Ago3, Siwi, HP1a or -b as indicated. Luciferase activities were measured at 72 h post-transfection. The luciferase activities were normalized to that of dsGFP-introduced controls (n = 3 independent luciferase activities). Error bars = SD. The SDs and *P*-values (*t*-test, *P<0.05, which was checked by comparing luciferase activities in dsGFP-introduced controls with each dsRNA against putative piRNA-related gene-introduced cells) are based on n = 3.
(TIF)

Table S1 The list of primers used in Supporting Materials and Methods.
(TIF)

Methods S1 Supporting Materials and Methods.
(PDF)
