# Supplementary figures and images for: Roles of Piwi Proteins in Transcriptional Regulation Mediated by HP1s in Cultured Silkworm Cells
Source: PLoS One. 2014 Mar 17;9(3):e92313. doi: 10.1371/journal.pone.0092313 (PMC3956929; doi:10.1371/journal.pone.0092313)

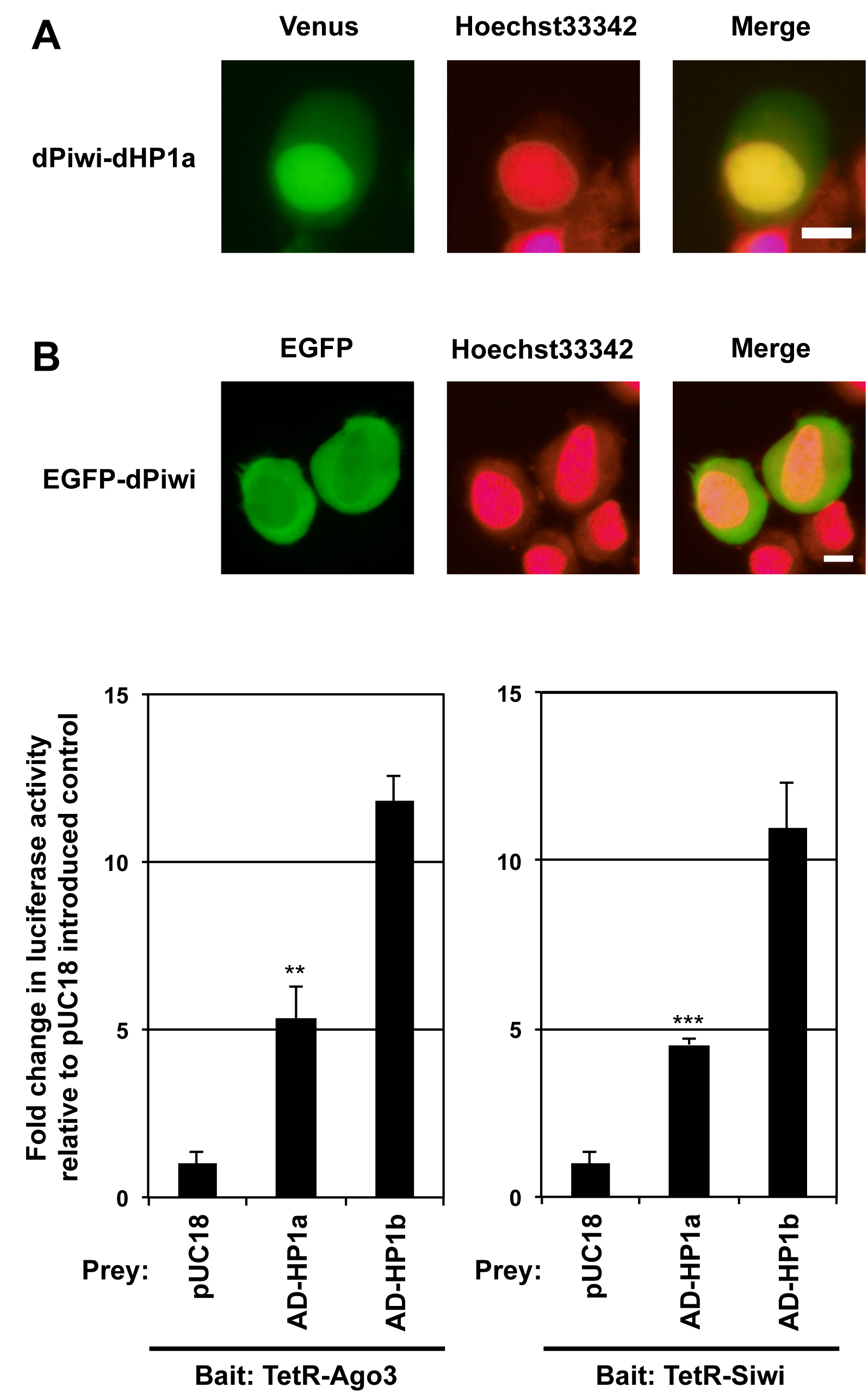

Supplement: Figure S1 — The interaction between Piwi and HP1 proteins in silkworm cultured cells. (A) The interaction between D. melanogaster Piwi and HP1a in BmN4 cells. The BiFC analysis for the interaction between Piwi and HP1 proteins in the silkworm cultured cell line BmN4. Venus and Hoechst33342 fluorescences indicate their localization and nucleus, respectively. Scale bar: 10 μm. (B) The localization of EGFP-fused Drosophila Piwi protein in a BmN4 cell. EGFP and Hoechst33342 fluorescences indicate their localization and nucleus, respectively. Scale bar: 10 μm. (C, D) The insect two-hybrid (I2H) assay for the interaction between the silkworm Piwi and HP1 proteins with the use of the TetR-fused Piwi proteins instead of GAL4 DNA-binding domain. BmN4 cells were transfected with 9×tetO-IE2mini(L)-Luc reporter construct, along with expression vectors for TetR and p65 activation domain (AD) fused to Ago3, Siwi, HP1a or HP1b as indicated. Empty cloning vector pUC18 was introduced as the control prey instead of pAD-HP1a or HP1b. Luciferase activities were measured at 72 h post-transfection. Values are relative luciferase activities in the pUC18-introduced control. Error bars = SD. The SDs and P-values (determined by the t-test, **P<0.01, ***P<0.001, which was checked by comparing luciferase activities in pUC18-introduced controls with pAD-HP1a or -b-introduced cells) are based on n = 3. (TIF) [file pone.0092313.s001.tif]

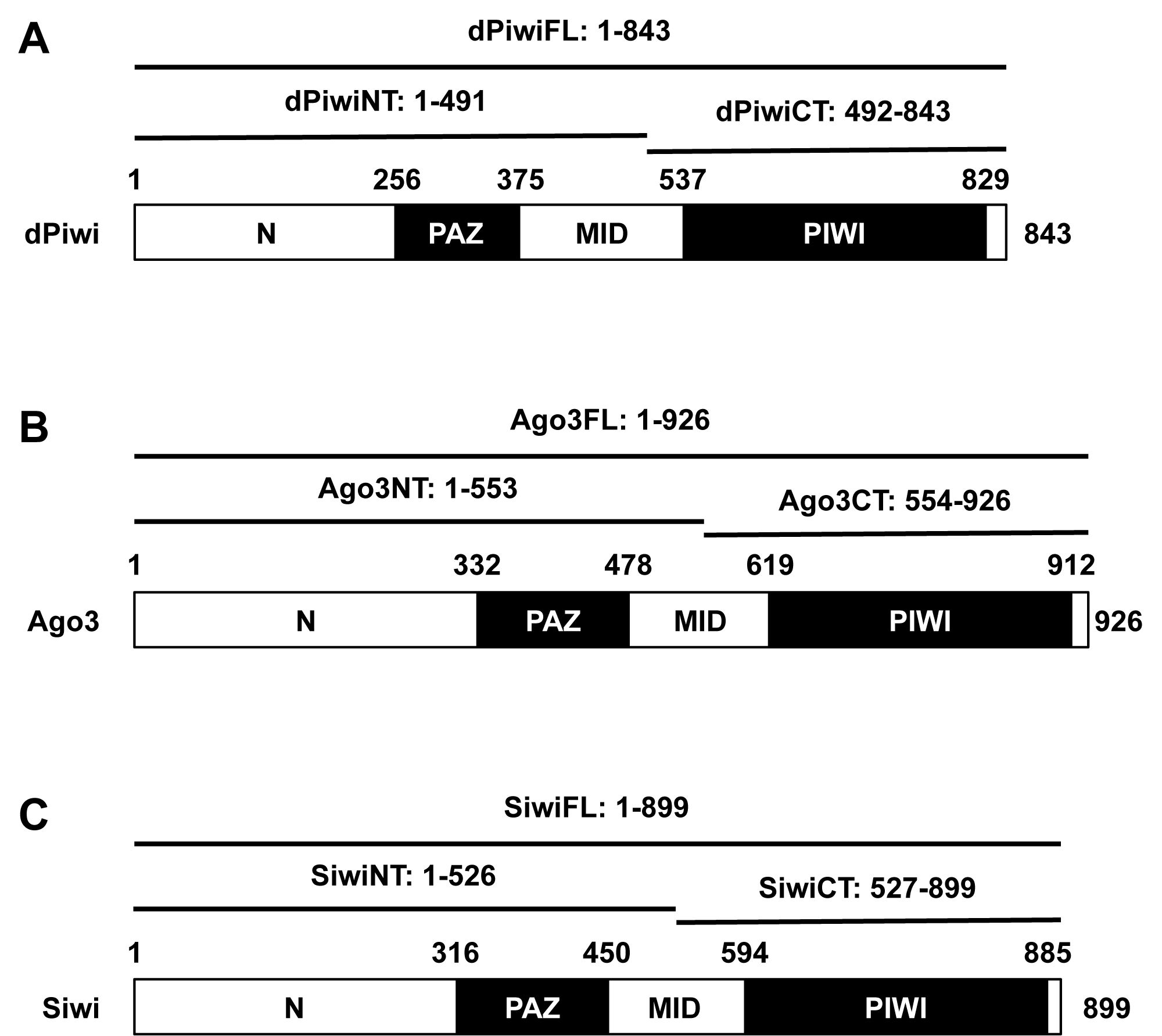

Supplement: Figure S2 — The split Piwi proteins for I2H baits. Schematic structure of baits used in the split Piwi I2H assay. Piwi protein contains N, PAZ, MID, and PIWI domains. (A) dPiwiFL, residues 1–843; dPiwiNT, residues 1–491; and dPiwiCT, residues 492–843. (B) Ago3FL, residues 1–926; Ago3NT, residues 1–553; and Ago3CT, residues 554–926. (C) SiwiFL: 1–899; SiwiNT: 1–526; and SiwiCT: 527–899. (TIF) [file pone.0092313.s002.tif]

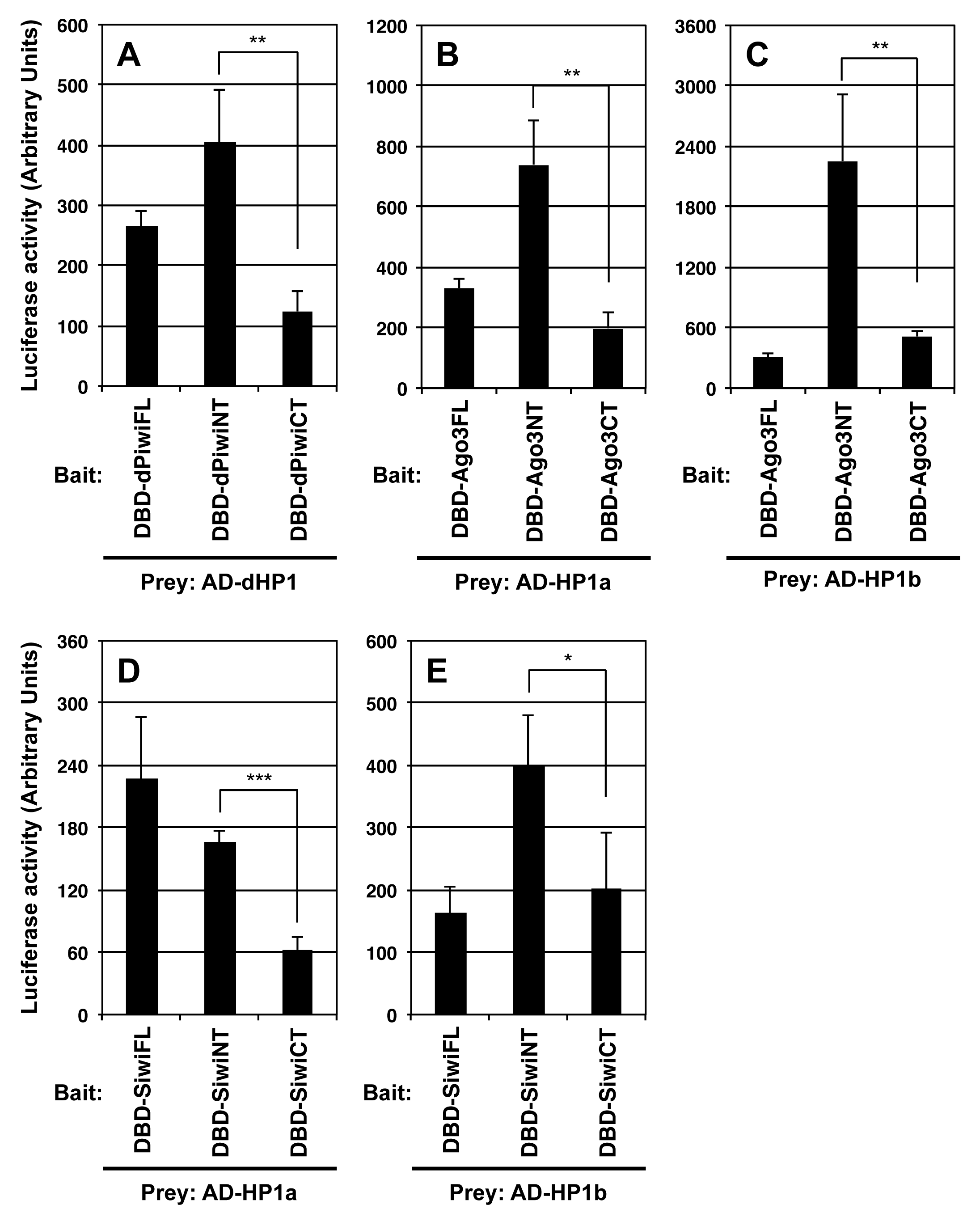

Supplement: Figure S3 — The N-terminal domain of silkworm Piwi proteins interacts more strongly with HP1a/b than does the C-terminal domain. (A–E) The I2H assay of the interaction between the split Piwi and HP1 proteins. BmN4 cells were transfected with 4×UAS-TATA-Luc reporter construct, along with expression vectors for GAL4 DNA-binding domain (DBD) and p65 activation domain (AD) fused to dPiwiFL, dPiwiNT, dPiwiCT, Ago3FL, Ago3NT, Ago3CT, SiwiFL, SiwiNT, SiwiCT, HP1a or HP1b as indicated. Luciferase activities were measured at 72 h post-transfection. Error bars = SD. The SDs and P-values (determined by the t-test, *P<0.1, **P<0.05, ***P<0.01, which was checked by comparing luciferase activities in cells transfected with DBD-fused N-terminal domain plasmids with that of DBD-fused C-terminal domain-transfected cells) are based on n = 3. (TIF) [file pone.0092313.s003.tif]

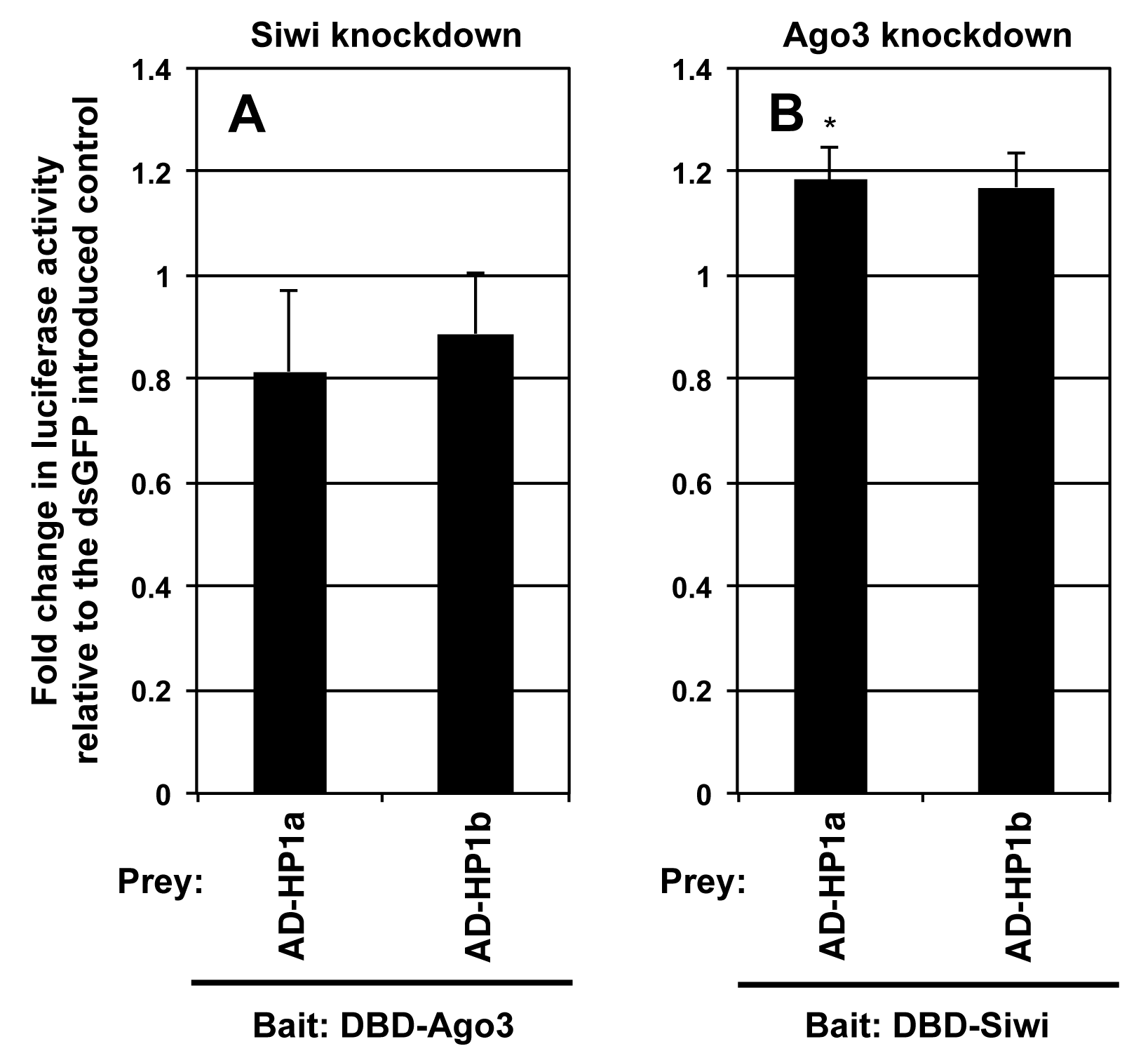

Supplement: Figure S4 — The I2H assay for the interaction between the silkworm Piwi and HP1 proteins in each of silkworm Piwi gene knocked-down BmN4-SID1 cells. (A, B) At 72 h after the introduction of dsAgo3, dsSiwi or dsGFP (control), BmN4-SID cells were transfected with 4×UAS-TATA-Luc reporter construct, along with expression vectors for GAL4 DNA-binding domain (DBD) and p65 activation domain (AD) fused to Ago3, Siwi, HP1a or -b as indicated. Luciferase activities were measured at 72 h post-transfection. The luciferase activities were normalized to that of dsGFP-introduced controls (n = 3 independent luciferase activities). Error bars = SD. P-values are shown in the bars. The SDs and P-values (t-test, which was checked by comparing luciferase activities in dsGFP-introduced controls with each of the dsRNAs against Ago3 or Siwi gene-introduced cells) are based on n = 2 (prey: AD-HP1b and bait: DBD-Ago3 in Siwi knocked-down BmN4-SID1 cells) and n = 3 (others). (TIF) [file pone.0092313.s004.tif]

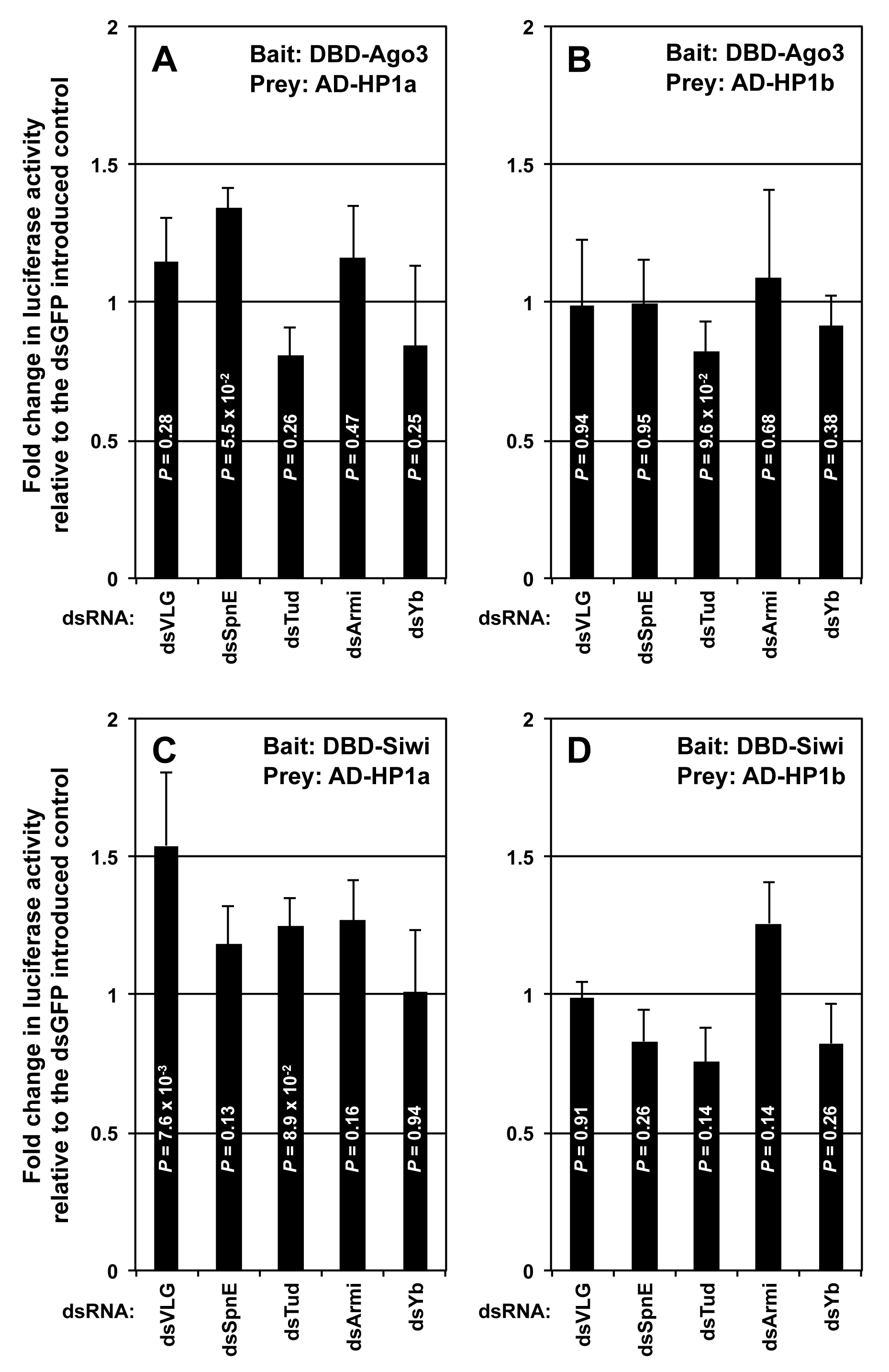

Supplement: Figure S5 — The I2H assay for the interaction between the silkworm Piwi and HP1 proteins in putative piRNA-related genes in knocked-down BmN4-SID1 cells. (A–D) At 72 h after the introduction of each dsRNA against putative piRNA-related genes, dsVLG, dsSpnE, dsTud, dsArmi, dsYb or dsGFP (control), BmN4-SID cells were transfected with 4×UAS-TATA-Luc reporter construct, along with expression vectors for GAL4 DBD and p65 AD fused to Ago3, Siwi, HP1a or -b as indicated. Luciferase activities were measured at 72 h post-transfection. The luciferase activities were normalized to that of dsGFP-introduced controls (n = 3 independent luciferase activities). Error bars = SD. The SDs and P-values (t-test, *P<0.05, which was checked by comparing luciferase activities in dsGFP-introduced controls with each dsRNA against putative piRNA-related gene-introduced cells) are based on n = 3. (TIF) [file pone.0092313.s005.tif]

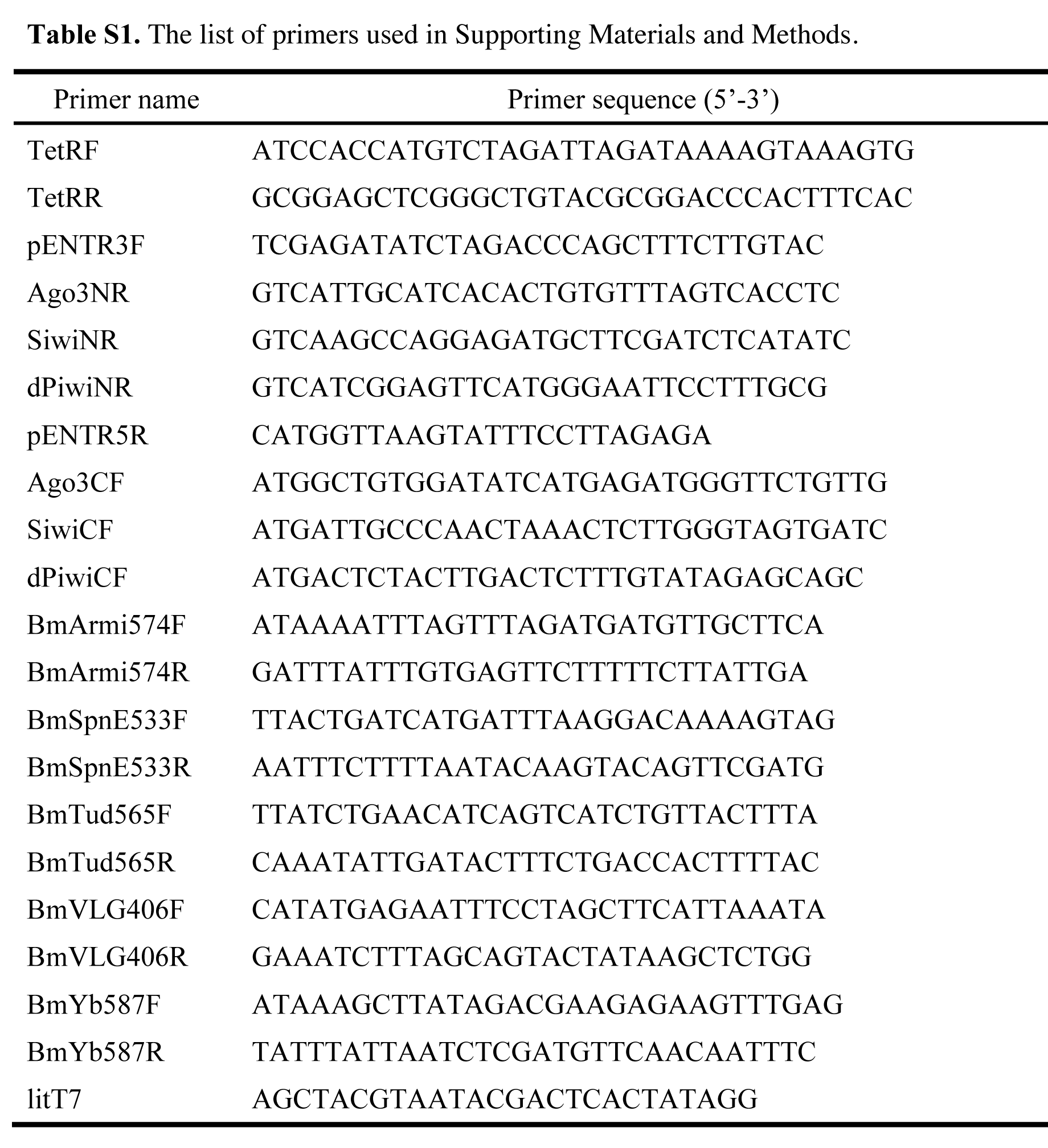

Supplement: Table S1 — The list of primers used in Supporting Materials and Methods. (TIF) [file pone.0092313.s006.tif]
