# Supplementary material for: Roles of Piwi Proteins in Transcriptional Regulation Mediated by HP1s in Cultured Silkworm Cells
Source: PLoS One. 2014 Mar 17;9(3):e92313. doi: 10.1371/journal.pone.0092313 (PMC3956929; doi:10.1371/journal.pone.0092313)
Supplement: Methods S1 — Supporting Materials and Methods. (PDF) [file pone.0092313.s007.pdf]

Supporting Information: Methods S1

Supporting Materials and Methods

Construction of plasmids

To generate EGFP fused dPiwi expression vector, the Gateway® LR reaction between the dHP1a-pENTR11 and dPiwi-pENTR11, and pie2GW, pnVW or pcCW [1, 2] was performed using LR Clonase™ Enzyme Mix (Invitrogen) according to the manufacturer's protocols recommended in manufacturer's manual. The resulting plasmids were named pEGFP-dPiwi, pCC-dHP1a and pNV-dPiwi.

To generate N-terminal fusion DEST vector, pIE2-TetR, for TetR-based I2H baits, *tetR* was amplified from pENTR11-NLS/TetR-Giant [2] by PCR using primers, TetRF and TeRR (Table S1). The PCR product was digested by *Sac*I and inserted into a *Stu*I-*Sac*I site of pie2FW [1]. The resulting plasmid was named pIE2-TetR. The Gateway® LR reaction between the entry vectors, BmAgo3-pENTR11 and Siwi-pENTR11, and the DEST vector, pIE2-TetR, was performed using LR Clonase™ Enzyme Mix (Invitrogen) according to the manufacturer's protocols recommended in manufacturer's manual. The resulting plasmids were pTetR-Siwi and pTetR-Ago3, which were used as bait expression vector for TetR-based I2H system together with prey expression vector and the tetO-based repoter, p9×tetO-IE2mini(L)-Luc [2].

The DNA sequence corresponding to N-terminal or C-terminal region of Piwi proteins were generated by inverse PCR from each entry clone, Ago3-pENTR11, Siwi-pENTR11 and dPiwi-pENTR11, using phosphorylated primers; for N-terminal

region coding entry clone, pENTR3F as the forward primer and the reverse primer for each gene, Ago3NR, SiwiNR and dPiwiNR; and, for C-terminal region coding entry clone, pENTR5R as the reverse primer and the reverse primer for each gene, Ago3CF, SiwiCF and dPiwiCF. The resulting plasmids were named Ago3NT-pENTR11, SiwiNT-pENTR11, dPiwiNT-pENTR11, Ago3CT-pENTR11, SiwiCT-pENTR11 and dPiwiCT-pENTR11, respectively. The Gateway® LR reaction between these entry vectors and the DEST vector, pIE2-DBD, was performed using LR Clonase™ Enzyme Mix (Invitrogen) according to the manufacturer's protocols recommended in manufacturer's manual. The resulting plasmids were pDBD-Ago3NT, pDBD-SiwiNT, pDBD-dPiwiNT, pDBD-Ago3CT, pDBD-SiwiCT and pDBD-dPiwiCT, which were used as bait for I2H assay.

Preparation of double-stranded RNA

Double-stranded RNA (dsRNA) for *in vivo* RNAi reactions was prepared using the following procedures. For the dsRNA template, BmArmi-574, BmSpnE-533, BmTud-565, BmVLG-406 and BmYb-587 fragments were amplified using primer pairs: BmArmi574F and BmArmi574R; BmSpnE533F and BmSpnE533R; BmTud565F and BmTud565R; BmVLG406F and BmVLG406R; and BmYb587F and BmYb587R, respectively (Table S1), from BmN4 cDNA library and were inserted into a *Stu*I site of pLits [3].

The templates for *in vitro* transcription were synthesized via PCR on the templates containing the BmArmi-574, BmSpnE-533, BmTud-565, BmVLG-406 and

BmYb-587 fragments using the litT7 primer (Table S1). The amplified DNA templates amplified were extracted with phenol/chloroform, precipitated with ethanol, and dissolved in H_2O. Bi-directional transcription reactions were performed in the buffer (40 mM Tris-HCl, pH 7.5, 6 mM $MgCl_2$, 10 mM DTT, 10 mM NaCl, 2 mM spermidine, 2 mM NTPs, 8 units of RNase inhibitor, and 20 units of T7 RNA polymerase), and incubated for 2 h at 37°C. The RNA products were purified and dissolved in 100 mM HEPES, pH7.0, incubated for 5 min at 94°C, and left to stand at room temperature for 30 min to allow the annealing of the two RNA strands, which are used as dsRNA solutions.

References

1. Mitsunobu H, Sakashita K, Mon H, Yoshida H, Lee JM, Kawaguchi Y, Koga K, Kusakabe T (2006) Construction of Gateway-based Destination Vectors for Detecting Subcellular Localization of Proteins in the Silkworm, *Bombyx mori*. J Insect Biotech Seric 75: 141-145

2. Li Z, Cheng D, Mon H, Tatsuke T, Zhu L, Xu J, Lee JM, Xia Q, Kusakabe T (2012) Genome-wide identification of polycomb target genes reveals a functional association of Pho with Scm in *Bombyx mori*. PLoS ONE 7: e34330

3. Tatsuke T, Lee JM, Kusakabe T, Iiyama K, Sezutsu H, Uchino K (2013) TIGHTLY CONTROLLED TETRACYCLINE-INDUCIBLE TRANSCRIPTION SYSTEM FOR EXPLOSIVE GENE EXPRESSION IN CULTURED SILKWORM CELLS. Arch Insect Biochem Physiol 82: 173-182

4. Li Z, Tatsuke T, Sakashita K, Zhu L, Xu J, Mon H, Lee JM, Kusakabe T (2012) Identification and characterization of Polycomb group genes in the silkworm, *Bombyx mori*. Mol Biol Rep 39: 5575-5588
